# Stereoptic serious games as a visual rehabilitation tool for individuals with a residual amblyopia (AMBER trial): a protocol for a crossover randomized controlled trial

**DOI:** 10.1186/s12886-023-02944-y

**Published:** 2023-05-17

**Authors:** Cristina Simon-Martinez, Maria-Paraskevi Antoniou, Walid Bouthour, Daphne Bavelier, Dennis Levi, Benjamin T. Backus, Brian Dornbos, James J. Blaha, Martina Kropp, Henning Müller, Micah Murray, Gabriele Thumann, Heimo Steffen, Pawel J. Matusz

**Affiliations:** 1 University of Applied Sciences Western Switzerland (HES-SO) Valais-Wallis, Rue de Technopole 3, 3960 Sierre, Switzerland; 2 Department of Ophthalmology, University Hospitals of Geneva, Geneva, Switzerland; 3 The Sense Innovation and Research Center, Lausanne and Sion, Sion, Switzerland; 4 Experimental Ophthalmology, University of Geneva, Geneva, Switzerland; 5 Faculty of Psychology and Education Sciences, University of Geneva, Geneva, Switzerland; 6 Herbert Wertheim School of Optometry & Vision Science, Helen Wills Neuroscience Institute, University of California Berkley, Berkley, CA USA; 7 Vivid Vision, Inc, 424 Treat Ave., Ste B, San Francisco, CA 94110 USA; 8 Institute of Health Sciences, School of Health Sciences, HES-SO Valais-Wallis, Sion, Switzerland; 9 Laboratory for Investigative Neurophysiology, Department of Radiology, Lausanne University Hospital, University of Lausanne (CHUV-UNIL), Lausanne, Switzerland; 10 Department of Hearing & Speech Sciences, Vanderbilt University Medical Center, Nashville, TN USA

**Keywords:** Amblyopia, Child, Binocular stimulation, Dichoptic stimulation, Optical treatment, Refractive correction, Virtual reality, Stereovision, Visual attention, Kinematics, Motor control, Electroencephalography

## Abstract

**Background:**

Amblyopia is the most common developmental vision disorder in children. The initial treatment consists of refractive correction. When insufficient, occlusion therapy may further improve visual acuity. However, the challenges and compliance issues associated with occlusion therapy may result in treatment failure and residual amblyopia. Virtual reality (VR) games developed to improve visual function have shown positive preliminary results. The aim of this study is to determine the efficacy of these games to improve vision, attention, and motor skills in patients with residual amblyopia and identify brain-related changes. We hypothesize that a VR-based training with the suggested ingredients (3D cues and rich feedback), combined with increasing the difficulty level and the use of various games in a home-based environment is crucial for treatment efficacy of vision recovery, and may be particularly effective in children.

**Methods:**

The AMBER study is a randomized, cross-over, controlled trial designed to assess the effect of binocular stimulation (VR-based stereoptic serious games) in individuals with residual amblyopia (*n* = 30, 6–35 years of age), compared to refractive correction on vision, selective attention and motor control skills. Additionally, they will be compared to a control group of age-matched healthy individuals (*n* = 30) to account for the unique benefit of VR-based serious games. All participants will play serious games 30 min per day, 5 days per week, for 8 weeks. The games are delivered with the Vivid Vision Home software. The amblyopic cohort will receive both treatments in a randomized order according to the type of amblyopia, while the control group will only receive the VR-based stereoscopic serious games. The primary outcome is visual acuity in the amblyopic eye. Secondary outcomes include stereoacuity, functional vision, cortical visual responses, selective attention, and motor control. The outcomes will be measured before and after each treatment with 8-week follow-up.

**Discussion:**

The VR-based games used in this study have been conceived to deliver binocular visual stimulation tailored to the individual visual needs of the patient, which will potentially result in improved basic and functional vision skills as well as visual attention and motor control skills.

**Trial registration:**

This protocol is registered on ClinicalTrials.gov (identifier: NCT05114252) and in the Swiss National Clinical Trials Portal (identifier: SNCTP000005024).

## Background

Amblyopia is one of the most common developmental vision disorders in children, affecting 1–5% of the population in developed countries [1, 2]. It arises from abnormal visual experience in early life. Amblyopia is most commonly caused by one or a combination of (i) significant refractive error (unilateral amblyopia caused by asymmetric error, or bilateral amblyopia caused by bilateral high refractive error); (ii) strabismus; or (iii) early visual deprivation (usually congenital causes) [3]. Besides significantly reduced visual acuity, amblyopic patients exhibit binocular dysfunction that may translate into reduced binocular reading speed [4], selective attention impairment [5], or motor control skills deficits [6].

Amblyopia is typically diagnosed around the age of 3–5 years, and the initial treatment consists of refractive correction [7]. If this measure fails to produce the desired outcome after 3 months, which is similar visual acuity between both eyes, occlusion therapy is commonly used [8]. This involves patching the dominant/healthy eye for 2–6 h/day, depending on the severity of the amblyopia, every day for several months up to years [9]. However, occlusion therapy carries the risk of reverse amblyopia and new strabismus by overtreatment [10]. In addition, poor adherence to the occlusion regimen is a common problem in pediatric populations (ranging from 49 to 87%), resulting in treatment failure [11] and residual amblyopia (i.e., reduced visual acuity and stereopsis that will persist into adulthood). Children with residual amblyopia may develop social and emotional problems (e.g., low self-esteem, bullying), which may affect their quality of life and that of their families [12, 13]. Therefore, it is important to seek suitable alternatives to the occlusion therapy.

Aiming to increase compliance and treatment effectiveness, serious games delivered on tablets have recently been developed (serious games are games used for purposes other than mere entertainment [14]). More specifically, these games focused on binocular stimulation by using dichoptic images, where the contrast level of the image to the fellow eye is reduced to encourage binocular integration of complementary images, and as such to balance cortical input and overcome interocular suppression. Such dichoptic stimulation delivered on tablets seems to effectively improve visual acuity [15–19] and might be superior to occlusion therapy [15, 20]. Nevertheless, the additional recovery of stereovision (3D vision) seems to require further ingredients in the training. Three studies have shown an improvement in stereovision in adults with amblyopia when using one game in a Virtual Reality (VR) environment [21–24], suggesting that a VR-based training providing 3D cues and rich feedback may better target improvement in stereovision in both strabismic and anisometropic type of amblyopia, improving also treatment compliance [25]. However, these preliminary studies in adults delivered the VR in a lab environment and with only one or two games. A recent review showed that binocular perceptual learning and dichoptic videogames result in improved stereovision in adults with strabismus, compared to the null results seen with monocular versions of such stimulation [26]. This review gathers data from almost 100 adults with amblyopia and provides three conclusions: (1) more patients with anisometropic amblyopia improve compared to the strabismic type, (2) many more patients with strabismus have not measurable stereopsis before and after the training, compared to the anisometropic type, and (3) both types of amblyopia show improvements in stereopsis, regardless of their baseline stereoacuity, achieving stereoacuities of 140 arcsec or better. As such, we hypothesize that a VR-based training with the suggested ingredients (3D cues and rich feedback), combined with increasing the difficulty level and the use of various games in a home-based environment is crucial for treatment efficacy of vision recovery, and may be particularly effective in children.

Besides the potential improvements in visual acuity and stereovision that the VR-training may induce, we also need to target the known selective attention and motor planning deficits in children with amblyopia. The VR environment offers an enriched, immersive (depth perception, stimulation of full visual field) perceptual experience, that puts strong demands on uni- (visual) and multisensory (audiovisual) selective attention, while performing natural body movements (reaching a target, body rotation to avoid enemy) [21, 27]. Hence, a VR environment with (i) a variety of available games that increases difficulty with improvement of function (to increase compliance), (ii) games that require time constraints and a high load on divided attention (to target selective attention, as shown in adults [28]) and (iii) the opportunity to interact with the body accurately to complete the tasks (to target motor deficits, as shown in stroke survivors [29]) appears to be an optimal training to improve selective attention and motor planning deficits associated with vision problems.

In addition to assessing the effectiveness of the novel treatment, this study may provide some mechanistic insight on visual improvement by exploring the neural substrates of vision, cognitive, and motor deficits in this population. With scalp electroencephalography (EEG), we can determine changes in the cortical activity before and after an intervention, in an easy and child-friendly way. EEG has been used to understand the visual deficits [30], to depict deficits of attentional modulation in the visual cortex [31, 32] and to understand the treatment-driven plasticity [33]. To the best of our knowledge, EEG has not been used to explore the sensorimotor networks in patients with amblyopia, although these networks seems to be affected, as revealed by other brain imaging techniques (i.e., resting state magnetic resonance imaging) [34]. These preliminary results provide a rationale for using EEG in amblyopia, to explore the plasticity of the cortical networks in residual amblyopia, and its relationship with clinical changes.

This study protocol describes the setup for a Randomized Controlled Trial (RCT) comparing the effects of a personalized, VR, and home-based binocular stimulation intervention in people with residual amblyopia compared to standard care and to healthy controls. The first objective is to examine whether VR game-based interventions are not inferior to refractive correction in residual amblyopia and whether it will result in similar or better retention of the improvements. Secondly, we aim to explore whether such VR-derived improvements in the amblyopic cohort are beyond the changes we find in the control cohort. Lastly, we will study the underlying neural mechanisms of improvements by measuring brain activity and will ascertain whether such mechanisms can predict, in combination with clinical measures, treatment outcome.

This RCT will address the following research aims:

- a) test the efficacy of a serious game-based binocular stimulation in a VR environment in improving Best Corrected Visual Acuity (BCVA), compared to refractive correction in individuals with residual amblyopia
- b) test the potential VR-based benefits on clinical measures of other vision skills like stereoacuity, functional vision (i.e., reading skills), and suppression, compared to refractive correction in individuals with residual amblyopia
- c) test the potential benefits of VR-based training on visual and audiovisual selective attention and motor control skills, compared to refractive correction and to the control group
- d) evaluate the adherence and safety of the VR-based intervention compared to standard care
- e) test the potential VR-based benefits on eye-related quality of life
- f) test the potential VR-based benefits on cortical responses including visual, higher cognitive processes of audio-visual, and motor control skills in both cohorts
- g) test whether the potential VR-derived benefits on vision, attention and motor control skills in residual amblyopic patients are larger from those found in the control cohort
- h) identify clinical and electrophysiological factors predicting treatment response in individuals with residual amblyopia.

## Methods I: Participants, interventions, and outcomes

### Study design

This is a single-center, evaluator-masked, non-inferiority, cross-over Randomized Controlled Trial (RCT). This study is approved by the Ethical Cantonal Board of Geneva, Switzerland (CCER N° 2021-D0090) and all methods are performed in accordance with the relevant guidelines and regulations. The protocol for this study was designed according to the SPIRIT (Standard Protocol Elements: Recommendations for Interventional Trials) 2013 Statement [35, 36]. Recruitment of participants is planned from May 2022 to December 2024. The trial will take place in the Department of Ophthalmology of the Geneva University Hospitals (Switzerland). The amblyopic cohort will take part in the cross-over design. They will be randomly assigned to take part either first in the study-intervention arm and second in the control-treatment arm, or vice versa (AB or BA), stratified according to the type of amblyopia (anisometropic, strabismic or mixed). They are not masked to the order of the received training. The age-matched control cohort will only complete the VR-based intervention.

### Participants and eligibility criteria

The study population will include 30 individuals with residual amblyopia (strabismus, anisometropic and mixed types) and 30 age-matched normally sighted individuals between 6–35 years old, as younger children seem to have difficulties in understanding the games [37]. General exclusion criteria are the presence of (i) auditory deficits or loss, (ii) uncorrected visual disorder, (iii) coexistence of ocular or neurological disease, and (iv) developmental, psychological, or sensorimotor disorder. Additional inclusion criteria for the amblyopic cohort are: (i) residual amblyopia defined as BCVA of < 20/20 in the amblyopic eye and an interocular difference of ≥ 2 lines persisting even after refractive correction; (ii) stable BCVA for at least 2 consecutive measurements over 6 months. Additional exclusion criteria for the amblyopic cohort are: (i) untreated or newly diagnosed anisometric, strabismic or combined amblyopia, i.e., a BCVA interocular difference of ≥ 2 lines that is untreated or newly diagnosed; (ii) atropine treatment currently or 3 months prior to enrolment in the study; (iii) eye surgery except those to correct strabismus; (iv) strabismus over 20D or with large eccentric fixation. In this study, as we include both healthy and amblyopic cohorts, we will use the terms dominant and non-dominant eye to refer also to the fellow and amblyopic eye, respectively.

### Interventions

#### Experimental intervention for the amblyopic cohort

The experimental intervention consists of playing eight stereoscopic serious games using the Vivid Vision Home software (Vivid Vision Inc., San Francisco, CA, USA), embedded in a VR headset. After a training session at the Dept. of Ophthalmology, the intervention is conducted at home, at the participant’s and family’s convenience. Participants’ success in the games depends on the integration of information from the amblyopic and the fellow eye, as the games are designed to improve binocularity. The games are aimed at training anti-suppression/fusion (Hoopie, Ring Runner, Breaker, Pepper Picker), stereopsis (Bubbles, Bullseye), and visual processing (Flash Match). Such game variability will improve the compliance among the older participants [37]. The prescribed dosage for game playing is 30 min per day, 5 days per week, over 8 weeks, with a total game time of 20 h. To prevent participants from playing more than 30 min per day, the software is programed to block access to its games until the next day. Participants can split the prescribed dosage of 30 min per day into smaller sessions, if they desire to do so. All participants will be instructed to wear their glasses with the updated refractive correction while they play the VR-based games.

At the beginning of the training, the visual contrast of objects visible to the fellow eye is decreased relative to the amblyopic eye. Each week and according to the participant’s performance, the difference in the input strength between the eyes will become smaller. The goal is to no longer need any modification of images’ features to combine them, and to perceive depth all the time. The difficulty of the games will be automatically adjusted based on an algorithm developed by Vivid Vision Inc. and integrated in the software (Smart Assist). This automatic adjustment aims at individualizing the treatment to meet the therapeutic needs of the patient. By individualizing the treatment to the patient’s needs, we hope to improve the efficacy of the treatment. To this purpose, built-in tests in the Vivid Vision Home software (Prism Tuner—to optimize the virtual prism settings for a patient’s treatment session, Stereoacuity – to estimate of the patient’s stereoscopic vision) will be performed systematically to increase or decrease the amount contrast needed for the patient to successfully play. These changes aim to keep the games challenging to the current level of visual skills of the participants. This “adaptiveness” of the games’ difficulty levels is meant to keep participants engaged and to support their improvement over the course of the whole training. Additionally, after each training session, participants or participants’ parents will report in a gaming diary, which they have been given by the experimenters, duration of their gaming session that day, their feelings about it (i.e., experiencing fun or, alternatively, difficulty during the game) as well as any adverse event (i.e., nausea, headache, diplopia). Participants will be instructed to inform the research team as soon as they experience any adverse event. Through the web-based dashboard of Vivid Vision Home, the research team will be able to see the participant’s activities, follow up on the prescribed play time and will get a notification after 2 days have passed without playing.

#### Control intervention for the amblyopic cohort

The control treatment for the amblyopic cohort involves wearing the prescribed glasses with the necessary refractive correction, which is the standard treatment for individuals who have undergone the patching treatment and been diagnosed with residual amblyopia. Participants will wear their glasses for the same duration as the experimental intervention (i.e., 8 weeks). The participants are expected to not receive any concomitant care or any additional interventions.

#### Control intervention for the control cohort

The control cohort will only undergo the VR-based intervention with the stereoscopic serious games on the Vivid Vision Home software. However, the games will not have any between-eyes image difference in terms of contrast (although those games with dichoptic stimulation will be displayed in the same way). The Smart Assist algorithm will be turned off for this cohort.

### Outcomes

Our primary outcome is best corrected visual acuity. Among our secondary outcomes are stereoacuity, functional vision (reading), visual cortical responses, higher cognitive processes of multisensory attention (i.e., visual and audiovisual attention), motor control skills, and eye-related quality of life. Primary and secondary outcome measures will be evaluated before and after each treatment as well as 8-weeks follow up (a more detailed timeline can be found later in this protocol).

#### Primary outcome

Best Corrected Visual Acuity (BCVA) will be measured using the Sloan chart adapted to age. The BCVA is the diagnostic vision measure for amblyopia (i.e., an interocular difference in visual acuity between the amblyopic and non-amblyopic eye) and it has been shown to improve with patching treatment as well as with novel treatments with binocular stimulation [17, 19, 38]. This will be administered by certified orthoptists who are masked to the treatment order.

#### Secondary outcomes I: clinical measures

##### Binocular vision: stereoacuity

Stereoacuity refers to the smallest detectable depth difference that can be seen in binocular vision. When binocular vision is present, the binocular function is the best stereoscopic acuity, measured in arc seconds. The presence of binocular vision will be first tested with the Bagolini. Stereoacuity will be measured with the Lang stereotest II, the TNO stereo test, and Titmus tests at every assessment point and the assessors are masked to treatment order.

Additionally, we will measure stereoacuity with a novel, 3D tablet-based test called ASTEROID [39] at every assessment point and the assessors are not masked to treatment order, as this is computerized test.

##### Binocular vision: interocular misalignment

Interocular misalignment refers to the degree to which two eyes' axes are not parallel. It can be measured with the Prims-cover test. Result of the Prism-cover test of one eye turning upon covering the other indicates eye misalignment. Red filter involves asking patient to fixate on a white circle at the end of the room and placing a red filter on the patient's fellow eye. If the patient reports a red pinkish light, there is binocular fusion. Additionally, it provides information on the alignment of the eyes based on normal retinal correspondence or binocular fusion based on abnormal retinal correspondence (e.g. microstrabismus). The location of the red circle in relation to the white circle lets conclude the nature of the deviation (esotropia, exotropia or vertical deviation). These tests are administered at every assessment point and the assessors are masked to treatment order.

##### Binocular vision: measurements in the Vivid Vision Home software

A Composite Depth Score estimate is measured (0–30) where 0 indicates no stereovision and 30 indicates a stereo threshold of 20 arc sec or better. Patients need to choose which of 4 circular stimuli are floating off the surface, where with each correct response the stimuli become smaller and the disparity decreases. The degree of binocular vision is estimated with a virtual Worth 4 Dot test, revealing normal vision, double vision, or suppression of left or right eye [40]. Ocular posture adjustment estimates the minimal correction needed for patient’s ocular posture in a horizontal, vertical and rotational prism is estimated in prism diopters through a Maddox rod like test [41], where the patient aligns vertical and horizontal lines with a spot or a pair of horizontal lines. Vergence is evaluated through the speed of the patient’s ability to switch between difference vergence demands is estimated in seconds as the participant is aligning a series of shapes or symbols until they make up a single line [42]. Lastly, the vergence range is the participant’s maximal horizontal and/or vertical vergence ability and is estimated in prism diopters as the participant is aligning a series of shapes or symbols until they make up a single line [42]. These tests are measured at every assessment point and are automatically performed by the software.

#### Secondary outcomes II: behavioral measures

##### Functional vision: reading skills

Reading skills will be measured, separately for each eye, using the standardized MNRead test [43]. This test is administered through an app on an iPad^©^ and is designed to assess reading skills in people with low vision (MNRead, French electronic version, 2016). The MNRead measures the smallest print readable by the person without making errors (e.g., misread or missing words, according to the MNRead instructions), as well as the smallest print that the person can read with maximum speed and the maximum reading speed. However, as fluent reading is necessary for this test, only children from 8 years old will perform it.

##### Selective attention processes

Visual and audiovisual selective attention will be measured with a selective-attention task, administered separately for each eye. The paradigm is a spatial cueing task of Folk et al. (1992) [44], adapted to multisensory settings and so that it is child-friendly [45, 46]. Participants search for a target diamond of a predefined color (e.g., blue) in an array of four differently colored bars and will report the bar’s orientation (i.e., horizontal or vertical) by pressing one of two large buttons. On every trial, the search array is preceded by a task-irrelevant visual distractor, appearing randomly in one of the four stimulus locations. The distractor is either of the same color as the target (e.g., blue), or of another, non-target color (e.g., red). On half of all trials the visual distractor is presented together with a spatially diffuse tone (audiovisual distractor). The target color will be counterbalanced across participants.

Two types of selective attention processes will be measured: top-down, goal-based visual attention and bottom-up, stimulus-driven multisensory attention. They will be assessed behaviorally and electrophysiologically (EEG), comparable to Folk et al. [44] and Turoman et al. [45, 46], respectively. Behaviorally, the two processes of selective attention will be measured using spatial cuing effects, i.e., the difference in speed of responding when the cue and target are in the same vs. different locations. Specifically, the strength of visual selective attention will be measured by the difference in cuing effects elicited by distractors that matched vs. mismatched the target’s color. The strength of audiovisual selective attention will be measured by the difference in cuing effects elicited by the color distractors that appeared alone vs. with the sound.

Motor control: Reaching and GRAsping at different Depths (ReGraD) Task.

Motor planning and execution deficits will be evaluated during a visually guided reach-to-grasp task at different reaching depths using reflective markers attached to the fingers and hand. The participant sits in front of a wooden box (where the pegs are initially inserted) and a cylinder (where the pegs are to be inserted). The box is mounted on a tripod that can be adjusted to the participant height and arm length. There are two equally sized pegs that are placed by the experimenter in the box differing by their color on the tip of the peg. The participant is instructed to focus on the target peg (green) and ignore the flanker peg (red). The participant needs to reach and grasp the target peg by estimating its depth and insert it in the cylinder. The board on the wooden box has 2 rows of 3 holes (right on top of each other) where the pegs can be inserted. The target and flanker pegs can be inserted in the same column, with 1 or 2 column difference, to increase depth between the pegs. There will be a total of 90 trials to complete with each eye condition (dominant eye, non-dominant eye and with both eyes):

- (i) No depth difference: 20 trials (5 trials with target in close-top position, 5 trials with target in further-top position, 5 trials with target in close-bottom position and 5 trials with target in further-bottom position). In this condition, the flanker does not influence stereovision perception.
- (ii) One level of depth difference: 20 trials (10 with the target in front (5 up and 5 bottom) and 10 (5 up and 5 bottom) with the flanker in front). In this condition, the flanker may influence stereovision perception either when it is located in front or after the target peg.
- (iii) Two levels of depth difference: 20 trials with two levels of depth difference (10 with the target in front (5 up and 5 bottom) and 10 with the flanker in front (5 up and 5 bottom)). In this condition, the flanker may influence stereovision perception either when it is located in front or after the target peg.
- (iv) Only target condition: 20 trials with only the target inserted in the same locations as in the ‘no depth difference’ condition. This is the control condition to investigate the potential effect of the flanker on the position of the target.

**Fig. 1:**
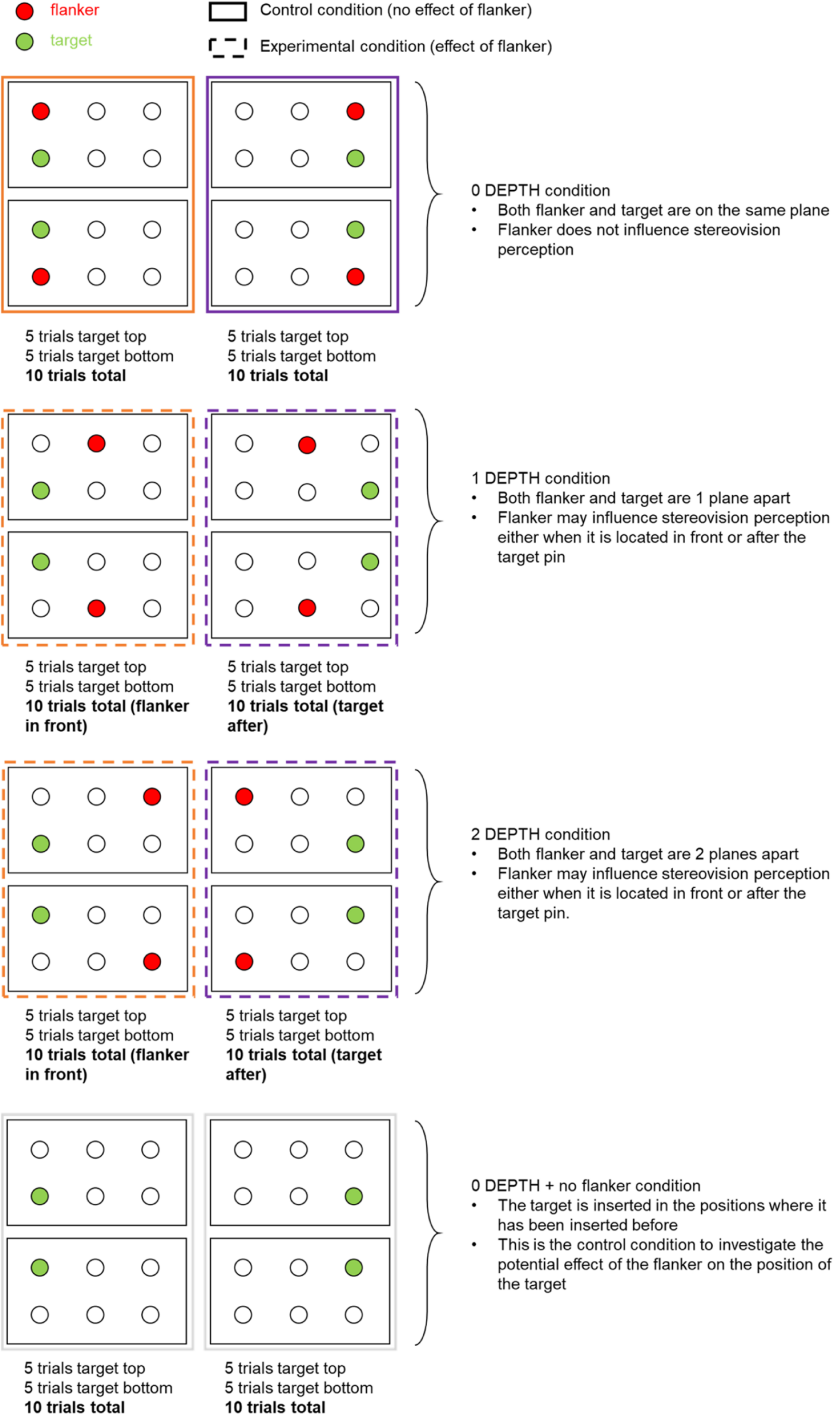
Possible configurations of the pegs used in the reaching and grasping at different depths task

A custom python script has been developed to show the experimenter where to insert the pegs for each trial, randomizing the order. The possible configurations can be seen in Fig. 1. The participant is instructed to open the eyes when they hear a ‘beep’ (when they see the peg configuration) and close them again once the peg is inserted in the cylinder.

To record the participant’s motion, we will use reflective markers attached to the tip of the index and thumb, on the head of the 3^rd^ metacarpophalangeal joint. An additional marker will be placed at the nasion, to monitor head motion. Lastly, a marker will be placed at each tip of the pegs. Participant’s motion will be recorded with a V120:Trio camera (OptiTrack NaturalPoint, USA).

We will split it into reaching phase, manipulating phase and withdrawal phase. From each phase, we will extract movement duration, reaction time, smoothness, maximum grip aperture and time to maximum grip aperture [47–50]. To evaluate the movement duration, we will calculate the time passed from the start to the end of the movement. To evaluate the reaction time, we will calculate the time passed from the start of the trial (indicated with a custom script) to the start of the movement. To evaluate the smoothness, we will extract the information from the marker placed on the hand and we will calculate its trajectory straightness. The straighter, the smoother the data are, which indicates better motor control. To evaluate the maximum grip aperture, we will extract the difference between the positions of the marker on the index and the marker on the thumb. The maximum difference will be used. To evaluate the time to maximum grip aperture, we will extract the difference between the positions of the marker on the index and the marker on the thumb. The time at which the maximum difference occurred will be used. This will be done for three conditions: monocular dominant, monocular non-dominant, and binocular.

##### Quality of life

Eye-related quality of life and functional vision in people with visual impairment will be assessed with the PedEyeQ questionnaire [13, 51], including one for the parents of participants younger than 14 years old and an adapted version for participants older than 18 years old. This questionnaire will not be completed by healthy individuals or their parents.

Rasch scores for each questionnaire item will be obtained from published look-up tables available at www.pedig.net, and used to calculate a score for each participant (Parent-PedEyeQ for < 18-year-olds; adapted Child-PedEyeQ for > 18-year-olds). Scores will also be converted to a 0–100 scale to aid in interpretation. The Child PedEyeQ version includes the fields Functional Vision, Bothered by Eyes and Vision, Social, Frustration / Worry. The Parent PedEyeQ version includes the fields Impact on Parent and Family, Worry about Child’s Eye Condition, Worry about Self-perception and Interactions, Worry about Functional Vision. These tests are measured at every assessment point.

#### Secondary outcomes III: Brain activity

We will use a 128-channel eWave + cap connected to an amplifier (ScienceBeam, Shenzen, China) to record continuous EEG during the selective attention and motor control tasks (1000 Hz sampling rate). Electrode impedances will be kept below 50 kΩ, and electrodes will be referenced to the Cz electrode. Offline filtering will involve a 0.1 Hz high-pass, 40 Hz low-pass, as well as a 50 Hz notch using a second-order Butterworth filter (–12 dB/octave roll-off, computed linearly with forward and backward passes to eliminate phase-shift). EEG will be next screened for transient noise, eye movements, and muscle artefacts using an automatic artefact rejection criterion of ± 100 μV for adults and ± 150 μV for children, along with visual inspection. Data from artefact contaminated electrodes across all groups will be interpolated using three-dimensional splines [52].

##### Event-related potential analyses

For event-related potential (ERP) analyses, after data cleaning, the EEG will be segmented into peri-stimulus epochs from 100 ms before stimulus onset to 500 ms after the stimulus onset. Subsequently, epochs will be averaged according to the relevant conditions, and baseline corrected. The sensory and attentional processes measured with ERPs will be analyzed with traditional analyses of ERP correlates of those mental processes (see below), followed by multivariate analysis of EEG activity elicited in the well-known time-windows of the said ERP correlates. Multivariate analyses of the EEG/ERPs focus on the reference-independent characteristics of the whole electrical field across the scalp. Here, the multivariate analyses will focus on two different measures. First, we will analyze if potential differences in EEG/ERPs across conditions and/or populations stem from modulations in the topographic EEG activity. Differences in the topography of EEG response forcibly indicate that different configurations of sources have been recruited during responses across conditions/populations of interest. The topographic analyses here will involve clustering of the group-averaged EEG/ERP activity over the time-window of the ERP components of interest. These analyses are aimed at identifying periods of stable topographic patterns (topographic maps) and differences therein across conditions of interest. After the selection of an optimal number of topographic patterns explaining the EEG data, the patterns are fit back onto single-subject data; parameters like the EEG pattern duration (or map onset, map offset) and global explained variance will be analyzed. Second, we will analyze, if the potential differences in the EEG/ERPs stem from modulations in the strength of brain responses across different conditions/populations. Global Field Power (GFP) is the standard deviation of the moment-by-moment voltage of the electrical field across the whole scalp and reflects differences in the strength of brain response, across two or more conditions, within a statistically indistinguishable brain network.

These outcomes will be measured at every time point and the assessors will not be masked to treatment allocation, as these are objective measures.

##### Cortical visual responses

Visual evoked potentials (VEPs) will be extracted from the continuous EEG measures to assay the time-locked response to the visual targets and by extension the integrity of the cortical visual processing pathway. VEPs are informative about the spatio-temporal brain dynamics of sensory and perceptual processes. They consist of a sequence of voltage peaks measured over the occipital electrodes: negative peak (N100), positive peak (P100), followed by a negative peak (N200). VEPs will be recorded, for each eye separately, from responses to the color targets in the selective attention task [53]. These target-elicited VEPs are large in both children (+ 4 years old) and adults, and as such should serve as robust EEG markers of the visual cortical pathway integrity related to the fellow/dominant and amblyopic/non-dominant eye in adults and children.

Integrity of the sensory visual processes will be measured by differences in 1) the sequence and/or duration of EEG patterns and 2) the strength of brain response, measured with GFP, elicited by the visual targets over the VEPs’ time-window across different eye conditions and populations.

##### Selective attention processes

Typically, EEG/ERP processes underlying selective attention are measured with the N2pc component, a traditional marker of attentional selection of target objects among distractors. The N2pc is a negative-going voltage deflection observed approx. 200–300 ms after presentation of the stimulus of interest, larger over electrodes contralateral than ipsilateral to the side of the stimulus. The strength of visual selective attention here will be measured by the difference in the mean amplitude of the N2pc elicited by distractors that matched vs. mismatched the color of the target. Respectively, the strength of audiovisual selective attention here will be measured by comparing the N2pc mean amplitude across distractors presented alone vs. with sound.

For multivariate analyses, the strength of different types of selective attention here will be measured by the difference in the duration of EEG patterns present over the N2pc time-window elicited by distractors that match vs. mismatch the target color (visual selective attention), and distractors presented alone vs. with sound (audiovisual selective attention), in line with our previous studies on the development of neurocognitive processes underlying audio-visual attention in healthy populations [32, 46, 53]. In turn, for the GFP analyses, strength of visual and audiovisual selective attention here will be measured by the difference in the GFP elicited over the N2pc time-window by distractors that match vs. mismatch the target color (visual selective attention), and distractors presented alone vs. with sound (audiovisual selective attention).

##### Oscillatory analyses: Motor control data

For this study, we are especially interested in the primary motor and visual cortices bilaterally. The communication between these two distant brain areas provides the basis for integration of complex information, that helps us adapt our movement to changes in the environment [54]. Besides these two main areas, visuomotor tasks involve the supplementary motor area, the primary sensory cortex, the premotor cortex and the parietal cortex [55, 56]. Given the number of involved areas, we will include all electrodes in the oscillatory analysis. Once the data are cleaned according to the abovementioned procedures, we will divide the data according to each movement segment (reach, manipulate, withdraw). An additional segment of interest is the pre-movement segment, that corresponds to 2000 ms before the GO signal and 1000 ms after (to include the first part of motion) [57]. The EEG and behavioral data will be synchronized post-hoc using timestamps.

We will perform time–frequency analysis to investigate the power at a specific frequency band during the different segments (or epochs) of the task execution (see below). Time–frequency analysis can characterize the temporal dynamics of three of the features of oscillations contained in the EEG data: frequency, power, and phase [58]. Time–frequency power and the phase synchrony will be computed. Time–frequency power has been used to link brain activity to a variety of cognitive and motor mechanisms. Phase synchrony provides information about the timing of the oscillations at a specific frequency and can be examined across trials to capture how consistent or synchronous the phase of the oscillations is across trials [58]. Additionally, this technique provides a close interpretation of the neurophysiological mechanisms. Time–frequency analysis measures the dynamic changes in amplitude and phase of neural oscillations across different frequencies [58]. In a recent study, time–frequency analysis has been shown to be able to depict increased connectivity between the visual and motor cortex during action observation, compared to connectivity between other brain areas, whilst other techniques did not detect such pattern [59].

In this investigation, we will explore the changes in alpha, beta, and gamma frequencies. Whilst alpha power seems to predict accuracy in visuomotor tracking tasks [55], changes in beta frequency are related to movement planning and execution [60]. Increased synchronization in the high gamma-frequency range has also been shown to be related to active movement initiation [61], which may provide additional insights on the cortical dynamics of motor control.

## Secondary outcomes IV: Adherence and safety

Adherence (total amount of training hours, regularity of training) throughout the treatment will be investigated and the visual outcome will be correlated to the adherence that is automatically recorded by the Vivid Vision Home system. By including these measures of adherence to the serious games intervention, we will obtain important information [62], as the traditional treatment is known to have low adherence [11].

At the end of the study, the frequency and type of adverse events will be analyzed and compared across groups by the researcher masked to group allocation.

### Participant timeline

**Fig. 2:**
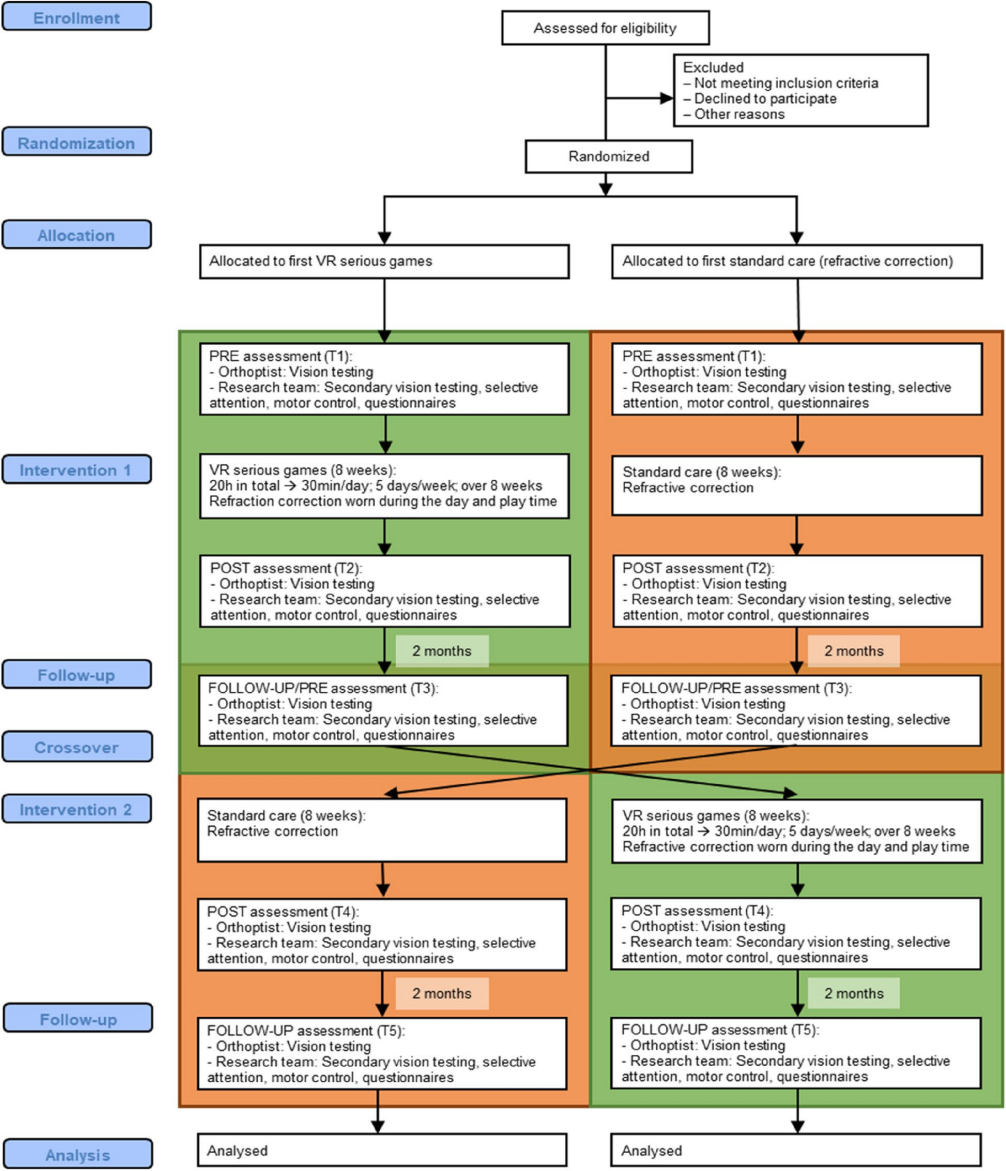
Flowchart of the study procedures and assessments for the amblyopic cohort. Left path would be the order AB and right path BA. Green represents the VR intervention and orange represents standard care intervention. Outcome measures are assessed immediately before and after each intervention and at 8-weeks follow-up

**Fig. 3:**
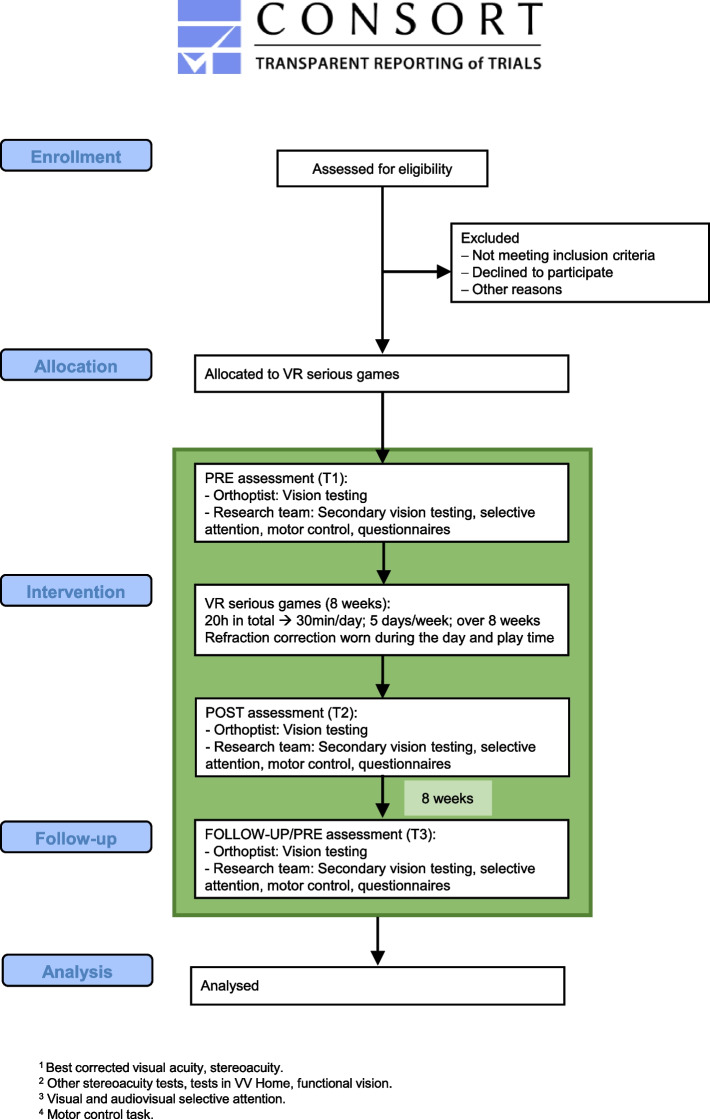
Flowchart of the study procedures and assessments for the control cohort. This cohort only completes the VR intervention. Outcome measures are assessed immediately before and after the intervention and at 8-weeks follow-up

The outcome measures will be measured before and after the intervention phase and 8 weeks follow up. Specifically for the amblyopic cohort, the outcome measures will be performed at 5 time points (Fig. 2): pre-training A (T1), post-training A (T2), 8-weeks follow up training A and pre-training B (T3), post-training B (T4), 8-weeks follow up training B (T5). For the control cohort, the outcomes will be measured at 3 time points (Fig. 3): pre-training (T1), post-training (T2), 8-weeks follow up (T3). Certified and experienced orthoptists (4 and 35 years of experience) will perform the assessments of the primary outcome measure (BCVA) and the basic-vision secondary outcome measures (see below). The orthoptists will be masked to the randomization, i.e., the order of the treatment (AB or BA). The remaining secondary outcomes (functional vision, selective attention, motor control) will be assessed by the research team. Before the start of the treatment, the research team will set up the VR games at the participant’s home. Participants will receive a manual with instructions on how to use the VR games, and access to a website with instructional videos.

### Sample size

We calculated the sample size necessary to obtain a significant difference (α = 0.05) between our treatment and refractive correction based on medium effect size (F’s Cohen = 0.25) with 80% power for a crossover design immediately after the intervention on our primary endpoint. This estimation resulted in a total sample of 24 individuals with residual amblyopia. To account for the potential dropout, we will increase the sample size by 25%, resulting in 30 individuals with amblyopia. We will additionally recruit 30 age-matched typically developing individuals to undergo the serious game VR-training. This sample will serve to account for the potential changes in vision, selective attention and motor control which are known to occur in healthy adults after a VR-training.

### Recruitment

The amblyopic cohort will be identified through referrals by ophthalmologists and optometrists from the Neuro-ophthalmology, Strabismus and Pediatric Ophthalmology Unit of the Dept. of Ophthalmology, HUG, and other private clinics. The control cohort will be recruited via printed flyers at public venues, schools, and colleagues’ children and friends. Recruitment will occur across the Geneva, Vaud, and Valais cantons. Participants will be offered to participate in the study once we are sure they meet all inclusion criteria without fulfilling any exclusion criteria. Before entering the study, written informed consent from all parents or caregivers and patients older than 14 years old will be obtained. Additionally, verbal assent will be obtained from participants between 6–14 years old. Through the insurance taken by the HES-SO Valais (sponsor of the study), the participants are insured for possible injuries as a result of their participation in the study.

## Methods II: Assignment of interventions

### Allocation

The amblyopic cohort will be randomly allocated to a treatment order (AB, BA) according to type of amblyopia (anisometropic, strabismic, mixed) and informed about the order by the research team. The allocation sequence will be generated with computer-generated random numbers, that will be created by an independent person of the project and uploaded to REDCap (concealed allocation with “sealed envelopes”). Only the research team will have access to the randomization instrument in REDCap. Whilst our sample size calculation is 30 participants in the amblyopic cohort (the control cohort will not be randomized), we have created a list with 10 additional cases. With this, in case we have unplanned dropouts, we will be able to continue with the original allocation list.

### Masking

The participants are not masked to the treatment order. Primary and secondary endpoint assessors of clinical data (orthoptists conducting the evaluation of the visual function), as well as the statistical analyst, will be masked to treatment order assignment. The research team, evaluating attention and motor function, are not masked as they also conduct the intervention. Nevertheless, these measures are objective and computerized, thus minimizing bias due to lack of masking.

## Methods III: Data collection, management, and analysis

### Data collection

All assessors will be trained to collect the study data. We have developed a handbook specific for the AMBER RCT in which all information related to the protocol is described and every study member has access to it.

The data will be collected at HUG (Geneva, Switzerland) and at the patients’ home (data collected directly from the Vivid Vision Home software and diary). General patient’s characteristics, such as age, sex and dominant eye will be recorded at baseline. Participants in the amblyopic cohort will be classified according to their type of amblyopia (anisometropic, strabismic or mixed).

All efforts will be made to promote participant retention and complete the crossover design of the study. We will phone the participants weekly while they are receiving the VR-based intervention and collect data related to their compliance and adverse events. All participants will fill in a diary in which they will report, if they have played the games of the intervention, as well as other games (and how many hours), to keep track of potential confounders. These data will be used to inform about participant retention in the study outcomes.

### Data management

All collected data will be coded and subsequently entered and stored in REDCap [63, 64], a secure, web-based application designed to support data capture for research studies by building and managing online surveys and databases.

### Statistical analysis

Linear Mixed Models (Jamovi & R software) will be used to investigate primary and secondary outcomes before and after treatment, compared to refractive correction, and compared to the healthy control group. We will include BCVA and stereoacuity measured at baseline and age as covariates. If age shows a significant covariance effect, we will identify clusters of age-based differences and compare the groups directly by splitting the data and using age as a factor in the analysis. For the selective attention skills, multivariate analyses will also be performed to investigate changes in brain activity (CARTOOL [65], STEN software [66], FieldTrip toolbox [67], custom scripts in Python). To evaluate motor control skills, trajectory and velocity profiles using Statistical Parametric Mapping [68] will be determined. Structural Equation Modelling will be conducted to identify the individual and combined value of behavioral and electrophysiological changes in vision, selective attention, and motor control to predict improvements in vision functions (Onyx software).

## Methods IV: Monitoring

Independent monitoring will be organized for the RCT in which all trial-related documentation will be checked in two visits: after the first 3 participants will have been recruited and after 20% (i.e., *n* = 6) of the participants will have been recruited. The monitor will check that the informed consents are signed, the adverse events forms are filled and up to date and that the data is entered in the electronic database (REDCap).

Whilst we do not expect the occurrence of adverse events, every time an adverse event occurs, the clinical team will be notified, and a decision will be made according to the severity of the adverse event and its potential relation to the treatment.

## Discussion

There is a need for innovative treatments for amblyopia that can increase patients’ compliance and additionally target stereovision and related cognitive skills like functional vision, attention, and motor control skills, garnered with a favorable risk–benefit ratio. In this study, we will determine the efficacy of binocular stimulation embedded in serous videogames in a VR environment as a home-based, child-friendly rehabilitation regime for residual amblyopia across children, adolescents, and young adults. The inclusion of brain activity measures may shed some light into the underlying mechanisms of improvement in each type of amblyopia, which will help the clinical field personalizing the treatments provided to each patient. As such, we will provide this important evidence, to pursue or abandon the approach in amblyopia treatment involving binocular treatment delivered in an engaging context.

## Data Availability

Not applicable.

## Appendix

Amblyopia: loss or lack of development of clear vision in one or both eyes.

Binocular vision: type of vision in which a person is able of using both eyes to perceive a single three-dimensional object.

Stereoacuity: visual acuity to perceive objects in three dimensions (length, width and depth), solely based on the relative positions of the objects in the two eyes.

Strabismus: condition in which both eyes do not look at the same place at the same time. It usually occurs in people who have poor eye muscle control or are very farsighted.

## Abbreviations

3D: Three-dimensional
AMBER: AMBlyopia and stEReovision
BCVA: Best Corrected Visual Acuity
EEG: Electroencephalography
ERP: Event-Related Potential
GFP: Global Field Power
HES-SO Valais: University of Applied Sciences and Arts Western Switzerland Valais)
HUG: Geneva University Hospitals
RCT: Randomized Controlled Trial
REDCap: Research Electronic Data Capture
ReGraD: REaching and GRAsping at different Depths
STEN: Statistical Toolbox for Electrical Neuroimaging
VEP: Visual Evoked Potentials
VR: Virtual Reality

## References

[1] Hashemi H, Pakzad R, Yekta A, Bostamzad P, Aghamirsalim M, Sardari S (2018). Global and regional estimates of prevalence of amblyopia: a systematic review and meta-analysis. Strabismus.

[2] Fu Z, Hong H, Su Z, Lou B, Pan C-W, Liu H (2020). Global prevalence of amblyopia and disease burden projections through 2040: a systematic review and meta-analysis. Br J Ophthalmol.

[3] Maurer D, McKEE SP (2018). Classification and diversity of amblyopia. Vis Neurosci.

[4] Webber AL (2018). The functional impact of amblyopia. Clin Exp Optom.

[5] Verghese P, McKee SP, Levi DM (2019). Attention deficits in Amblyopia. Curr Opin Psychol.

[6] Grant S, Moseley MJ. Amblyopia and real-world visuomotor tasks. In: Strabismus. 2011. p. 119–28. 10.3109/09273972.2011.600423 21870915

[7] Holmes JM, Clarke MP (2006). Amblyopia Lancet.

[8] Stewart CE, Moseley MJ, Fielder AR (2011). Amblyopia therapy: an update. Strabismus.

[9] Li T, Qureshi R, Taylor K (2019). Conventional occlusion versus pharmacologic penalization for amblyopia. Cochrane Database Syst Rev.

[10] Patil PA, Meenakshi S, Surendran TS (2010). Refractory reverse amblyopia with atropine penalization. Oman J Ophthalmol.

[11] Vagge A, Nelson LB (2017). Compliance with the prescribed occlusion treatment for amblyopia. Curr Opin Ophthalmol.

[12] Holmes JM, Beck RW, Kraker RT, Astle WF, Birch EE, Cole SR (2004). Risk of amblyopia recurrence after cessation of treatment. J AAPOS.

[13] Hatt SR, Leske DA, Castañeda YS, Wernimont SM, Liebermann L, Cheng-Patel CS (2020). Understanding the impact of residual amblyopia on functional vision and eye-related quality of life using the PedEyeQ. Am J Ophthalmol.

[14] Laamarti F, Eid M, El Saddik A (2014). An overview of serious games. Int J Comput Games Technol.

[15] Gambacorta C, Nahum M, Vedamurthy I, Bayliss J, Jordan J, Bavelier D (2018). An action video game for the treatment of amblyopia in children: a feasibility study. Vision Res.

[16] Birch EE, Li SL, Jost RM, Morale SE, De La Cruz A, Stager D (2015). Binocular iPad treatment for amblyopia in preschool children. J AAPOS.

[17] Holmes JM, Manh VM, Lazar EL, Beck RW, Birch EE, Kraker RT (2016). Effect of a binocular iPad game vs part-time patching in children aged 5 to 12 years with amblyopia. JAMA Ophthalmology.

[18] Vedamurthy I, Nahum M, Huang SJ, Zheng F, Bayliss J, Bavelier D (2015). A dichoptic custom-made action video game as a treatment for adult amblyopia. Vision Res.

[19] Kelly KR, Jost RM, Wang Y-Z, Dao L, Beauchamp CL, Leffler JN (2018). Improved binocular outcomes following binocular treatment for childhood amblyopia. Invest Ophthalmol Vis Sci.

[20] Hess RF (2022). Reasons why we might want to question the use of patching to treat amblyopia as well as the reliance on visual acuity as the primary outcome measure. BMJ Open Ophth.

[21] Vedamurthy I, Knill DC, Huang SJ, Yung A, Ding J, Kwon OS (2016). Recovering stereo vision by squashing virtual bugs in a virtual reality environment. Philos Trans R Soc Lond B Biol Sci.

[22] Žiak P, Holm A, Halička J, Mojžiš P, Piñero DP (2017). Amblyopia treatment of adults with dichoptic training using the virtual reality oculus rift head mounted display: Preliminary results. BMC Ophthalmol.

[23] Godinez A, Martín-González S, Ibarrondo O, Levi DM (2021). Scaffolding depth cues and perceptual learning in VR to train stereovision: a proof of concept pilot study. Sci Rep.

[24] Li RW, Tran KD, Bui JK, Antonucci MM, Ngo CV, Levi DM (2018). Improving adult amblyopic vision with stereoscopic 3-dimensional video games. Ophthalmology.

[25] Li L, Xue H, Lai T, Xue Y, Luo G (2022). Comparison of compliance among patients with pediatric amblyopia undergoing virtual reality-based and traditional patching method training. Front Public Health.

[26] Levi DM, Knill DC, Bavelier D (2015). Stereopsis and amblyopia: a mini-review. Vision Res.

[27] Levin MF, Demers M (2021). Motor learning in neurological rehabilitation. Disabil Rehabil.

[28] Green CS, Bavelier D (2003). Action video game modifies visual selective attention. Nature.

[29] Eng K, Siekierka E, Pyk P, Chevrier E, Hauser Y, Cameirao M (2007). Interactive visuo-motor therapy system for stroke rehabilitation. Med Biol Eng Compu.

[30] Kiorpes L, Daw N (2018). Cortical correlates of amblyopia. Vis Neurosci.

[31] Hou C, Kim Y-J, Lai XJ, Verghese P (2016). Degraded attentional modulation of cortical neural populations in strabismic amblyopia. J Vis.

[32] Mortazavi M, Aigner K, Antono JE, Gambacorta C, Nahum M, Levi D, et al. Neural correlates of visual spatial selective attention are altered at early and late processing stages in human amblyopia. Eur J Neurosci. 2020;:ejn.15024-ejn.15024. 10.1111/ejn.15024 33107117

[33] Shi W, He L, Lv B, Li L, Wu T (2020). Evaluating the acute effect of stereoscopic recovery by dichoptic stimulation using electroencephalogram. Comput Math Methods Med.

[34] Lin X, Ding K, Liu Y, Yan X, Song S, Jiang T (2012). Altered spontaneous activity in anisometropic amblyopia subjects: revealed by resting-state FMRI. PLoS ONE.

[35] Chan A-W, Tetzlaff JM, Altman DG, Laupacis A, Gøtzsche PC, Krleža-Jerić K (2013). SPIRIT 2013 Statement: Defining Standard Protocol Items for Clinical Trials. Ann Intern Med.

[36] Chan A-W, Tetzlaff JM, Gøtzsche PC, Altman DG, Mann H, Berlin JA (2013). SPIRIT 2013 explanation and elaboration: guidance for protocols of clinical trials. BMJ.

[37] Kadhum A, Tan ETC, Levi DM, Colpa L, Fronius M, Simonsz HJ (2021). Barriers to successful dichoptic treatment for amblyopia in young children. Graefes Arch Clin Exp Ophthalmol.

[38] Bossi M, Tailor VK, Anderson EJ, Bex PJ, Greenwood JA, Dahlmann-Noor A (2017). Binocular therapy for childhood amblyopia improves vision without breaking interocular suppression. Invest Ophthalmol Vis Sci.

[39] Vancleef K, Serrano-Pedraza I, Sharp C, Slack G, Black C, Casanova T (2019). ASTEROID: a new clinical stereotest on an autostereo 3D tablet. Transl Vis Sci Technol.

[40] Worth 4 Dot. Vivid Vision. https://www.seevividly.com/info/Binocular_Vision/Vision_Tests/Worth_4_Dot. Accessed 22 Jan 2023.

[41] Maddox rod. Wikipedia. 2022.

[42] Gall R, Wick B, Bedell H (1998). Vergence facility: establishing clinical utility. Optom Vis Sci.

[43] Calabrèse A, Owsley C, McGwin G, Legge GE (2016). Development of a reading accessibility index using the MNREAD acuity chart. JAMA Ophthalmol.

[44] Folk CL, Remington RW, Johnston JC (1992). Involuntary covert orienting is contingent on attentional control settings. J Exp Psychol Hum Percept Perform.

[45] Turoman N, Tivadar RI, Retsa C, Murray MM, Matusz PJ (2021). Towards understanding how we pay attention in naturalistic visual search settings. Neuroimage.

[46] Turoman N, Tivadar RI, Retsa C, Maillard AM, Scerif G, Matusz PJ (2021). Uncovering the mechanisms of real-world attentional control over the course of primary education. Mind Brain Educ.

[47] Simon-Martinez C, Mailleux L, Jaspers E, Ortibus E, Desloovere K, Klingels K (2020). Effects of combining constraint-induced movement therapy and action-observation training on upper limb kinematics in children with unilateral cerebral palsy: a randomized controlled trial. Sci Rep.

[48] Simon-Martinez C, dos Santos GL, Jaspers E, Vanderschueren R, Mailleux L, Klingels K (2018). Age-related changes in upper limb motion during typical development. PLoS ONE.

[49] Melmoth DR, Grant S (2012). Getting a grip: different actions and visual guidance of the thumb and finger in precision grasping. Exp Brain Res.

[50] Grant S, Conway ML (2019). Some binocular advantages for planning reach, but not grasp, components of prehension. Exp Brain Res.

[51] Leske DA, Hatt SR, Wernimont SM, Castañeda YS, Cheng-Patel CS, Liebermann L (2021). Quality of life and functional vision across pediatric eye conditions assessed using the PedEyeQ. J AAPOS.

[52] Perrin F, Pernier J, Bertnard O, Giard MH, Echallier JF (1987). Mapping of scalp potentials by surface spline interpolation. Electroencephalogr Clin Neurophysiol.

[53] Turoman N, Tivadar RI, Retsa C, Maillard AM, Scerif G, Matusz PJ (2021). The development of attentional control mechanisms in multisensory environments. Dev Cogn Neurosci.

[54] Hummel FC, Gerloff C, Neuper C, Klimesch W (2006). Interregional long-range and short-range synchrony: a basis for complex sensorimotor processing. Progress in Brain Research.

[55] Rilk AJ, Soekadar SR, Sauseng P, Plewnia C (2011). Alpha coherence predicts accuracy during a visuomotor tracking task. Neuropsychologia.

[56] Van Impe A, Coxon JP, Goble DJ, Wenderoth N, Swinnen SP (2011). Age-related changes in brain activation underlying single- and dual-task performance: Visuomanual drawing and mental arithmetic. Neuropsychologia.

[57] Struber L, Baumont M, Barraud P-A, Nougier V, Cignetti F (2021). Brain oscillatory correlates of visuomotor adaptive learning. Neuroimage.

[58] Morales S, Bowers ME (2022). Time-frequency analysis methods and their application in developmental EEG data. Dev Cogn Neurosci.

[59] Debnath R, Salo VC, Buzzell GA, Yoo KH, Fox NA (2019). Mu rhythm desynchronization is specific to action execution and observation: evidence from time-frequency and connectivity analysis. Neuroimage.

[60] Barone J, Rossiter HE (2021). Understanding the Role of Sensorimotor Beta Oscillations. Front Syst Neurosci.

[61] Cheyne D, Ferrari P (2013). MEG studies of motor cortex gamma oscillations: evidence for a gamma “fingerprint” in the brain?. Front Hum Neurosci.

[62] Gao TY, Black JM, Babu RJ, Bobier WR, Chakraborty A, Dai S (2021). Adherence to home-based videogame treatment for amblyopia in children and adults. Clin Exp Optom.

[63] Harris PA, Taylor R, Minor BL, Elliott V, Fernandez M, O’Neal L (2019). The REDCap consortium: building an international community of software platform partners. J Biomed Inform.

[64] Harris PA, Taylor R, Thielke R, Payne J, Gonzalez N, Conde JG (2009). Research electronic data capture (REDCap)—a metadata-driven methodology and workflow process for providing translational research informatics support. J Biomed Inform.

[65] Brunet D, Murray MM, Michel CM (2011). Spatiotemporal analysis of multichannel EEG: CARTOOL. Comput Intell Neurosci.

[66] Knebel J-F, Notter MP. Sten 1.0: statistical toolbox for electrical neuroimaging. 2012.

[67] Oostenveld R, Fries P, Maris E, Schoffelen J-M (2011). FieldTrip: open source software for advanced analysis of MEG, EEG, and invasive electrophysiological data. Comput Intell Neurosci.

[68] Pataky TC (2012). One-dimensional statistical parametric mapping in Python. Comput Methods Biomech Biomed Engin.

